# Uptake, Accumulation and Toxicity of Silver Nanoparticle in Autotrophic Plants, and Heterotrophic Microbes: A Concentric Review

**DOI:** 10.3389/fmicb.2017.00007

**Published:** 2017-01-26

**Authors:** Durgesh K. Tripathi, Ashutosh Tripathi, Shweta, Swati Singh, Yashwant Singh, Kanchan Vishwakarma, Gaurav Yadav, Shivesh Sharma, Vivek K. Singh, Rohit K. Mishra, R. G. Upadhyay, Nawal K. Dubey, Yonghoon Lee, Devendra K. Chauhan

**Affiliations:** ^1^Centre of Advanced Study in Botany, Banaras Hindu UniversityVaranasi, India; ^2^Center for Medical Diagnostic and Research, Motilal Nehru National Institute of Technology AllahabadAllahabad, India; ^3^D. D. Pant Interdisciplinary Research Laboratory, Department of Botany, University of AllahabadAllahabad, India; ^4^Department of Biotechnology, Motilal Nehru National Institute of Technology AllahabadAllahabad, India; ^5^Department of Physics, Shri Mata Vaishno Devi UniversityKatra, India; ^6^Lawrence Berkeley National LaboratoryBerkeley, CA, USA; ^7^Veer Chand Singh Garhwali Uttarakhand University of Horticulture and ForestryTehri Garhwal, India; ^8^Department of Chemistry, Mokpo National UniversityMokpo, South Korea

**Keywords:** nanotoxicology, silver, uptake, autotrophic plants, heterotrophic microbes

## Abstract

Nanotechnology is a cutting-edge field of science with the potential to revolutionize today’s technological advances including industrial applications. It is being utilized for the welfare of mankind; but at the same time, the unprecedented use and uncontrolled release of nanomaterials into the environment poses enormous threat to living organisms. Silver nanoparticles (AgNPs) are used in several industries and its continuous release may hamper many physiological and biochemical processes in the living organisms including autotrophs and heterotrophs. The present review gives a concentric know-how of the effects of AgNPs on the lower and higher autotrophic plants as well as on heterotrophic microbes so as to have better understanding of the differences in effects among these two groups. It also focuses on the mechanism of uptake, translocation, accumulation in the plants and microbes, and resulting toxicity as well as tolerance mechanisms by which these microorganisms are able to survive and reduce the effects of AgNPs. This review differentiates the impact of silver nanoparticles at various levels between autotrophs and heterotrophs and signifies the prevailing tolerance mechanisms. With this background, a comprehensive idea can be made with respect to the influence of AgNPs on lower and higher autotrophic plants together with heterotrophic microbes and new insights can be generated for the researchers to understand the toxicity and tolerance mechanisms of AgNPs in plants and microbes.

## Introduction

The ever-increasing indiscriminate anthropogenic activities worldwide together with the technological advances have led to the creation of huge waste material contaminating our biosphere and causing many ecological risks. Due to this, environmental stability is gradually diminishing thereby resulting in the damage to ecosystem facilities. In addition, the uncontrolled rise in human population will continue to intensify the ecosystem degradation in the near future (Lee, 2011). Due to the imbalanced population growth and simultaneous increase in ecological risk, the problems of food security and proliferation of pathogenic organisms may increase. Many scientists and pharmaceutical industries are working to develop antibacterial agents that can confer resistance against the attack of pathogens (Rai et al., 2009; Ahmed et al., 2013). In order to provide food for the increasing population, scientists are exploring new ways to increase the yield of the crops with the help of biotechnological techniques (Moose and Mumm, 2008). Presently, nanotechnology has proved to be an important tool in many industrial and agricultural applications such as raising productivity of many crops. The agricultural productivity can be increased by using nano-fertilizers or nanoparticles (NPs) in order to reduce the toxic effects of many metal pollutants (Anjum et al., 2013; Tripathi et al., 2015, 2016, 2017a). The naturally occurring NPs have always existed in the environment without any undesired properties (Murr et al., 2004; Handy et al., 2008; Macken et al., 2012). There are various modes of synthesis of Nps which include physical, chemical and biological methods. These smallest objects are referred to as the engineered NPs and may be counted as a whole unit in terms of its physiochemical or microscopic properties with a reduction of any one dimension (Donaldson and Poland, 2013). Such particles exhibit different behavior from their larger counterparts when reduced to nanoscale (Choi et al., 2008; Khanna, 2016). The production of engineered NPs will likely to increase from 2000 tons in 2004 to over 58,000 tons annually between 2011 and 2020 (Khanna, 2016). There are different varieties of nanoparticles and among them, silver nanoparticles (AgNPs) are fetching more attention because of their application or requirement in daily life (Chen and Schluesener, 2008; Aziz et al., 2015, 2016) as well as their toxic behavior (Tripathi et al., 2017b). In order to search for better solutions to the problems related to food security and occurrence of diseases, nanosilver is gaining priority as one of the leading solutions with more stability and surface area as compared to other nano-solutions (Donaldson and Poland, 2013; Khanna, 2016). Apart from this, AgNPs have wide range of applications in solar energy (Clavero, 2014), Raman scattering (Samal et al., 2013; Zheng et al., 2015), and antimicrobial applications (Rai et al., 2009). The effective antimicrobial properties and low toxicity of AgNPs toward mammalian cells have made them to be easily utilized in many consumer-based products. The silver nanoparticles also finds its use in biocidal coatings, shampoo, soap and toothpaste (Rai et al., 2009).

Owing to the increasing commercial production of NPs and their unregulated release into aquatic as well as terrestrial systems via number of pathways, there is a growing concern over their impending environmental effects (Choi et al., 2008; Mirzajani et al., 2013; Shweta et al., 2016; Singh et al., 2016). In a study by Nowack et al. (2011), it was observed that the potential concentration of AgNPs have increased in surface water up to 0.1 mg L^-1^ and in sludge up to 2.9 mg kg^-1^. Despite its beneficial applications, numerous harmful effects of AgNPs have also been reported in plants and animals (Navarro et al., 2008; Tripathi et al., 2017a,b). The eﬄuents having AgNPs are found to contaminate water bodies, soil and atmosphere (Benn and Westerhoff, 2008; Farkas et al., 2011; Nair and Chung, 2014). Cytotoxicity, genotoxicity and ecotoxicity of coated AgNPs have also been reported (Lee et al., 2007; Lima et al., 2012). It poses undesirable effects on plants such as inhibition of seed germination and growth (Yin et al., 2012; Dimkpa et al., 2013; Nair and Chung, 2014). From soil and water, they may penetrate into food crops (Mazumdar and Ahmed, 2011; Nair and Chung, 2014) and enter into heterotrophs or consumers by means of food chain. Studies have revealed that AgNPs show toxic behavior against mitochondria and generate reactive oxygen species (ROS) (Hsin et al., 2008; Kim et al., 2012). These ROS damage the cell membrane, disrupt ATP production pathway and DNA replication and alter gene expression (Moreno-Garrido et al., 2015). In algae and microbes also, it induces imbalanced generation of ROS and cause oxidative stress. There are various methods by which the affected plant or other organisms try to cope up with the problems induced by the NPs. Number of defense strategies are found in the organisms through which they avoid or lessen the possible impact of AgNPs. These defense mechanisms are important to understand as it may provide an exact understanding toward the amelioration of the problems arising due to the nanoparticle pollution and its impact on environment. However, the effect as well as the tolerance may vary across the organisms. The autotrophs show different response as compared to heterotrophs against NPs; thereby making it essential to understand such differences and related survival mechanisms. Hence, the present review details about the impact and tolerance of widely used nanomaterial, i.e., AgNP on both autotrophs and heterotrophs. It will lead to the enhancement of the knowledge in this regard and provide a differential approach towards the issue.

### Sources of Silver Nanoparticle in the Environment

**FIGURE 1:**
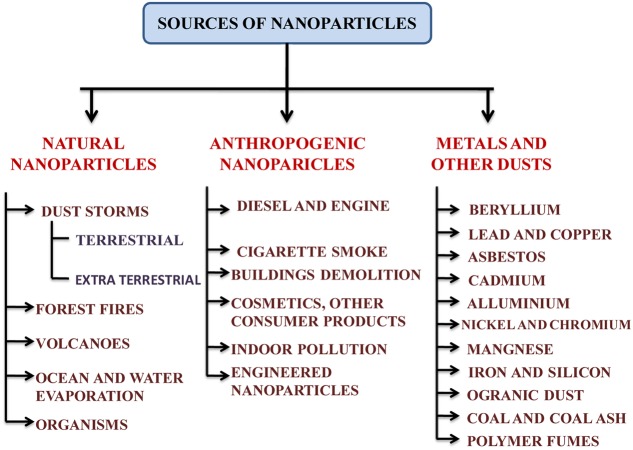
**An outline of the various sources of nanoparticles in the environment**.

Engineered NPs may be found in the form of metals, other dust or various compounds where they are used (**Figure 1**). Synthesis of the NPs in laboratory or industry is one of the important sources of its release in the environment (Bhaduri et al., 2013). Physical and chemical methods of NP synthesis are not eco-friendly and may contaminate the surrounding environment (Bhaduri et al., 2013; Kuppusamy et al., 2015) whereas biological synthesis of NPs is rather eco-friendly (Bhaduri et al., 2013). By using strong reducing and stabilizing agents, the chemical methods have an undesirable effect on biotic components (Bhaduri et al., 2013; Kuppusamy et al., 2015). However, the NPs synthesized from plant extract do not include any reductants or stabilizing agents (Carlson et al., 2008; Kuppusamy et al., 2015). An outline of the various point and non-point sources of AgNPs has been given in **Figure 1**.

The sources of metallic AgNPs are not new. AgNPs could have been naturally occurred via natural reduction process from Ag^+^ ions or produced anthropogenetically and then released into the environment (Nowack et al., 2012; Samal et al., 2013). Colloidal AgNPs had been produced and used as biocidal material in USA in 1954 (Nowack et al., 2011). The formation of AgNPs can be facilitated by photochemical reduction of Ag^+^ ions by dissolved organic matter in natural water under sunlight within several hours (Buzea et al., 2007; Samal et al., 2013). AgNPs may also be generated from silver objects through oxidative dissolution and subsequent reduction (Samal et al., 2013; Khanna, 2016). During washing, recycling, disposal and other manufacturing processes, they enter the surrounding environment (Nowack et al., 2011; Khanna, 2016) (**Figure 1**). The nanosilver species such as Ag^0^, AgCl, and Ag_2_S are frequently observed in various environmental compartments (Buzea et al., 2007; Wang et al., 2015; Khanna, 2016). There are various sources of AgNPs in the environment which could be point and non-point sources (Anjum et al., 2013) (**Figure 1**). AgNPs generated from anthropogenic activities are of greater concern as they are most widely incorporated in multidisciplinary applications. AgNPs are released in soil either from point sources that are suspended in surrounding environment after the application of NPs or organic matters in the forms of nano-fertilizers, sludge recycling, etc. in agricultural fields or from non-point sources such as products that contain AgNPs in themselves and directly contaminate the system (Mueller and Nowack, 2008; Benn et al., 2010).

Though AgNPs are found naturally, there should be no doubt that anthropogenic activities play a major role in pollution of silver nanoparticles in the environment. The widespread industrial uses of AgNPs have raised the chances of contamination. They are used in electronic devices, incorporated into textiles, dressing and medical devices, or directly added into disinfectants from where they could be directly released into the environment (Buzea et al., 2007; Khaydarov et al., 2009; Khanna, 2016). AgNPs may also appear from inappropriate disposal of biosolids or wastes, spills and other organic fertilizers or pesticides (Calder et al., 2012; Anjum et al., 2013). Despite these facts, the properties of AgNPs still enable them to be used in more than 250 consumer products in the world (Rai et al., 2009; Anjum et al., 2013). It is estimated that around 500 tons per annum AgNP is being produced (Mueller and Nowack, 2008), and is rapidly growing every year (Boxall et al., 2007). The generation of AgNPs in United States has been reported to be up to 2,500 tons per year from which approximately 150 ton is released in sewage sludge and 80 ton in surface waters (Khaydarov et al., 2009; El-Temsah and Joner, 2012).

### Chemistry of Silver Nanoparticles

In the periodic table, Silver (Ag) is an element of group 11 and period 5 with atomic number 47 and standard atomic weight 107.862. It has high electrical and thermal conductivity and also the reflectivity. This is considered as one of the main property of any metal. Silver belongs to the ‘d’ block in periodic table and its electronic configuration is [Kr]4*d*^10^5*s*^1^. It occurs in solid form with 2162°C and 961.78°C boiling and melting points, respectively. The density of silver is approximately 10.49 g/cm^3^, its oxidation state is +1 and atomic radius about 145 pm.

The range of AgNPs lies between 1 and 100 nm (Graf et al., 2003) that contains around 20–15,000 silver atoms (Anjum et al., 2013). However, the bulk material of silver may be silver oxide NP and characterized by high ratio of their surface area to bulk silver atom. Beside this, AgNP has distinct chemical and physical properties such as catalytic activity and non-linear optical characteristics. They are also found in different shapes and sizes such as spherical, octagonal or in the shape of sheets (Graf et al., 2003), rod shaped, cylindrical shaped, wire like, plate like, and belt like etc. (Pal et al., 2007; Jana et al., 2012; Kim et al., 2012; Anjum et al., 2013). Furthermore, they have various dimensions such as, zero dimensions, one dimension (1D), two dimension (2D), and three dimension (3D) and accordingly they may be laments, surface films, strands and particles, respectively (Tiwari et al., 2012). AgNP can be characterized by different spectrophotometric and electroscopic techniques such as SEM (Scanning Electron Microscopy), TEM (Transmission Electron Microscopy), XRD (X-ray Diffractometer), and UV-VIS spectrophotometer.

However, Mura et al. (2015) reported that AgNP can become more hazardous when oxidized in water because they make bonds with anions and hence transform into the characteristics of heavy metals. This conversion of AgNP to a complex of anion or heavy metal causes toxic effect on various living organisms (Chen and Schluesener, 2008; Wijnhoven et al., 2009; Fabrega et al., 2011; Anjum et al., 2013). Another distinct trait of AgNPs is large surface area-to-volume ratio, on the basis of which they act as antibacterial agent on both types of bacteria, i.e., Gram-positive and Gram-negative (Kim et al., 2007; Marambio-Jones and Hoek, 2010; Anjum et al., 2013).

### Applications of Silver Nanoparticles

Silver nanoparticles are intensively used in our daily life. AgNPs along with various other engineered NPs have wider application in many commercial and industrial sectors. It has also been used in the field of bioremediation and biomedicine because of its characteristic physiochemical properties (Chaloupka et al., 2010; Wong and Liu, 2010). Notably, they are used in antibiotics such as nanogels and nanolotions (Ma et al., 2010; Piccinno et al., 2012). These AgNPs are largely used in bedding, washers, toothpaste, waste water treatment, shampoo and fabrics, food packaging materials, food storage containers, water purifiers, odor-resistant socks and undergarments, room sprays, laundry detergents, etc. (Wijnhoven et al., 2009). Among other domestic uses, they are highly utilized for cleaning the bacteria from vacuum cleaner, refrigerators and ACs, laboratory coats, plastics, paints, textiles and other medical related applications such as in bandages, surgical gowns, wound dressings, female-hygiene products, bone cements and implantable devices etc. (Boxall et al., 2007; Kim et al., 2007; Klaine et al., 2008; Ma et al., 2010).

Due to some unique properties, AgNPs are used in sensing and imaging applications, including the detection of DNA (de la Escosura-Muñiz and Merkoçi, 2014), selective colorimetric sensing of cysteine, sensing purine nucleoside phosphorylase activity and selective colorimetric sensing of mercury(II) as well (Silver, 2003; Sapsford et al., 2013). Due to its antimicrobial activity, it inhibits the growth of both Gram-positive and Gram-negative bacteria and also its antibacterial activity is important for different drug-resistant pathogens (Samberg et al., 2011). Nanosilver is also used as an efficient fungicide against several ordinary fungal strains, such as *Aspergillus fumigatus, Mucor, Saccharomyces cerevisiae, and Candida tropicalis* (Velmurugan et al., 2009; Kim et al., 2012). AgNP also has antiviral properties which can be used against the HIV, hepatitis B and Herpes simplex virus (Galdiero et al., 2011). These are also used in many diagnostic and theranostic applications, such as in making nano-probes (Zheng et al., 2015; Li et al., 2016). However, we must understand that why silver nano differs from other nanomaterial in these applications. For example, gold NPs (AuNPs) are also widely used in medical science owing to their flow in endocytosis; they are diffused through lipid bilayer of the cell membrane and are mostly used in cancer treatments (Siddhanta et al., 2015; Alaqad and Saleh, 2016). Due to large surface-to-volume ratio, AuNPs functionalized with target specific biomolecules can efficiently destroy cancer cells or bacteria (Wang et al., 2010). AgNPs are commonly used due to their electrical conductivity, wide antimicrobial activity against various microorganisms and localized surface plasmon resonance effect (Raghavendra et al., 2014).

### Autotrophic Plants and Heterotrophic Microbes and Their Importance

Autotrophs are organisms that produce organic compounds (carbohydrates, fats, and proteins) from simple substances present in the surrounding by using energy from sunlight via photosynthesis. They are plants on land or algae in water. Autotrophs can reduce CO_2_ to make organic compounds and use water as the reducing agent, but some of them can also use other hydrogen compounds such as hydrogen sulfide for this purpose. However, the heterotrophs are organisms which are dependent on the autotrophs and cannot make their own food by fixing carbon rather they use organic carbon for their growth (Crane and Grover, 2010). The reduced carbon compounds in autotrophs provide the energy in food consumed by heterotrophs. All animals, fungi, most of the bacteria and protists are heterotrophs. Both kind of organisms have their own importance in an ecosystem in maintaining the food chain in which producers generate energy which is consumed by the consumers to degrade the organic compounds into simpler form to be free in the environment to complete biogeochemical cycles. Any change in the physiology and biochemistry of these organisms can, thus, disrupt the ecological balance in many ways (Crane and Grover, 2010). Hence, it is important to understand the impacts pose by any such chemical pollutant which is new to the environment and for which more elaborative studies are needed to regulate their release to the environments. The nanosilver is widely used nowadays and regularly released therefore; its uptake, accumulation, and toxicity must be known with respect to autotrophic and heterotrophic organisms in order to better understand the impact of nano-pollution and to search future ways to combat the problems.

## Interaction of Silver Nanoparticle with Autotrophs

### Interaction with Algae

Algae are considered as polyphyletic eukaryotic autotrophs which include many unicellular as well as multicellular forms and most of them are aquatic in nature and instead of lacking different tissues and cells like xylem and phloem, they make their own food. As most of the AgNP traces are released into the water after being used and are also employed for waste water treatment, it affects aquatic organisms in which algae are prime (Boxall et al., 2007). The toxicity of AgNPs toward algae can be estimated by means of many laboratory-based experiments and these studies demonstrate that AgNP is toxic to algae at different concentrations (Marshall et al., 2005; He et al., 2012; Moreno-Garrido et al., 2015). Due to different and variable growing conditions of these organisms, the amount of experiments and data on toxicity on various algal species are still sporadic. Since algal communities are important not only for the aquatic photosynthesis and food resource (Marshall et al., 2005) but also for industrial applications (Moreno-Garrido et al., 2015), therefore understanding the toxicity of nanosilver on this vital organism becomes necessary.

#### Uptake, Translocation, and Accumulation

The uptake, translocation, and accumulation of the AgNPs in the cells depend on the cellular structure, its permeability, size of the particles and other cell properties (Carlson et al., 2008; Li et al., 2015). The cell wall in algae is an important point for any type of reciprocal action with AgNPs as it acts as an obstruction or blocking point of the inflowing AgNPs from surrounding environment. The algal cell wall mainly comprise of carbohydrates, proteins, and cellulose (glycoproteins and polysaccharides) which organize a stiff elusive network (Navarro et al., 2008). Due to this, algal cell wall works as a semi permeable sieve and screens out larger NPs by allowing the transition of the smaller particles (Navarro et al., 2008). The smaller size and larger surface area of the AgNPs enable them to transit through the pores of cell wall and eventually reach to the plasma membrane (Samberg et al., 2011). Cellular reproduction may alter the permeability of cell wall and recently fabricated pores may become permeable for silver nanoparticles to a greater extent (Ovećka et al., 2005; Navarro et al., 2008).

It has been reported that due to the influence of AgNPs on algal cell, newly formed pores are larger than the prior ones and this may led to instigate the increase in uptake of the nanosilver in the cells of algae (Navarro et al., 2008). The sizes of pores in cell wall through which a single NP can be passed ranges from 5 to 20 nm. However, the interaction with NPs creates new and large-size pores in the cell wall and hence increases the internalization efficiency of cell (Carlson et al., 2008). After this transition through cell wall, AgNPs converge with plasma membrane. The possible mode of entry by lipid bilayer membrane has been discussed by some researchers (Navarro et al., 2008; Leonardo et al., 2015). AgNPs can encompass in cavities like the structure fabricated by plasma membrane and then can be imbibed into the cell through endocytic processes (Ovećka et al., 2005; Siddhanta et al., 2015). Apart from these, the ion channels or transport carrier proteins could also be used by AgNP as a mode of entry into the cell membrane (Mueller and Nowack, 2008). After entering into the cell, these NPs get attached with the various cell organelles (e.g., endoplasmic reticulum, Golgi bodies and endo-lysosomal system) and it shows some significant symptoms such as swelling of the endoplasmic reticulum and vacuolar changes (Miao et al., 2010). Navarro et al. (2008) reported that algal cell wall contains some barriers to create hindrance as well as some primary sites for interaction with NPs. Moreover, their bimolecular system contains many functional groups such as hydroxyl, carboxylate, imidazole, sulfhydryl, phosphate, and amine which are associated with many active sites of the AgNP interaction (Cao and Liu, 2010). After reaching to the specific cell organelle, they start disturbing the metabolic processes by enhancing the production of ROS and affect the biochemical processes in the cell (Miao et al., 2010).

#### Toxicity

Silver nanoparticles induces physical and chemical substructure alterations by means of its toxicity in the algal cells (**Table 1**). AgNP shows toxic effects as it releases silver ions and poses adverse effects on algal community at varied concentrations. The structural and functional properties of the algal cell could be affected by severe alterations induced by these NPs. The toxicity is induced through decrease in chlorophyll content, viable cell counts, increased ROS generation and lipids peroxidation (MDA) (Marshall et al., 2005; Miao et al., 2010; Dewez and Oukarroum, 2012; He et al., 2012; Li et al., 2015). It was noticed that AgNPs in association with light alter the oxygen evolution complex, inhibit the electron transport activity as well as induce some structural deterioration (He et al., 2012; Oukarroum et al., 2012; Leonardo et al., 2015). There are reports showing increased toxicity of AgNPs as compared to metallic silver ions which means silver ions are more toxic if present in the form of NPs in environments (Roh et al., 2009; Fabrega et al., 2010). The negative impact of AgNPs are also seen on the algal reproduction as well as on the subsequent stimulated imposition of oxidative stress (Roh et al., 2009; Fabrega et al., 2010; Ribeiro et al., 2014).

**Table 1:** Inimical effects of silver nanoparticles (AgNPs) on different algal varieties.

Algae	Size of AgNPs	Concentrations	Effect of NPs	Reference
*Chlamydomonas reinhardtii*	10 nm	10, 50, 100, and 500 μM	Reduction in photosynthetic yield of algae	Navarro et al., 2008
*Ceramium tenuicorne*	<5, 5–10 nm	26.6 μg L^-1^	AgNPs induce toxic effects in organism	Macken et al., 2012
*Chlorella vulgaris, Dunaliella tertiolecta*	50 nm	0–10 mg L^-1^	Strong decrease in chlorophyll content as well as formation of ROS and lipid peroxidation takes place	Oukarroum et al., 2012
*Pseudokirchneriella subcapitata*	20–30 nm	LC_50_ 0.19 mg L^-1^	Low toxicity of AgNPs observed than silver ions	Griffitt et al., 2009; Fabrega et al., 2010
*Chlamydomonas reinhardtii*	25 ± 13 nm	EC_50_ 1H: 3300 nM; EC_50_ 5h: 829 nM	Toxicity of silver ions observed released from AgNp accumulated in cell.	Navarro et al., 2008 Fabrega et al., 2010
*Thalassiosira weissflogii*	60–70 nm	0.02–0.0002 nM	Decreased production of chlorophyll and low photosynthesis rate. Reduced cell growth observed.	Fabrega et al., 2010
*Chara vulgaris*	10–15 nm	0.9 mM	Green colored thalli turned yellow due to progressive loss of chlorophyll	Das et al., 2012
*Pithophora oedogonia*	10–15 nm	1.5 mM	Fragmented and disintegrated chloroplasts; thin and ruptured cell wall; condensed and clumped chromosomes at metaphase stage	Das et al., 2012
*Ochromonas danica*	1–10 nm	More than 10 μM	Showed inhibiting effect even after supplementation of glutathione	Miao et al., 2010
*Thalassiosira weissflogii*	60–70 nm	0.02–0.0002 nM	Suppressed chlorophyll production, photosynthetic activity and hence growth of the cell	Miao et al., 2009
*Pseudokirchneriella subcapitata*	80 nm	Nominal EC_50_- 5.25 ± 1.82	Growth inhibited in size dependent manner	Ivask et al., 2014
*Chlorella* sp.	<100 nm	10 ppm	Shown to cause adverse effect on chloroplasts and finally death of cells	Zaidi et al., 2014
*Chlamydomonas acidophila*	50 nm	1, 10, and 100 mg L^-1^	Altered chlorophyllous contents, cellular and parameters like cellular viability, generation of intracellular ROS	Oukarroum et al., 2014
*Chattonella marina*	50 nm	10 μM	Generation of ROS	He et al., 2012

Various properties of the released Ag^+^ ion (such as preparation, stability, aggregation, and speciation) have differing impacts on algae (**Table 1**). Burchardt et al. (2012) have also demonstrated this difference in *Thalassiosira pseudonana* and *cyanobacterium Synechococcus*. Various algal species have been tested for the toxicity of AgNPs at various concentrations (**Table 1**) such as, *Dunaliella tertiolecta* and *Chlorella vulgaris* (Oukarroum et al., 2012), *T. pseudonana* and *cyanophyte Synechococcus* (Burchardt et al., 2012) and *Euglena gracilis* (Li et al., 2015). While Ribeiro et al. (2014) compared the effects and found that AgNPs were more toxic than silver nitrate. AgNPs have been reported to enhance the biotic generation of superoxide in *Chattonella marina* (He et al., 2012). The AgNPs also affect photosystem II (PSII) photochemistry, alternation of the oxygen evolution complex, inhibition of electron transport activity, and structural deterioration of PSII reaction of the green algal species (Navarro et al., 2015; Huang et al., 2016). AgNP acts as a catalyst for redox reactions when they get in touch with organic molecules and they also affect photosynthetic and respiratory processes (Navarro et al., 2008) which is an outcome of the impacts on photo-induced electron transfer capacity by AgNP (Navarro et al., 2008).

In various algal species, the toxicity mechanisms for AgNPs depend on various processes occurring in the cell such as adhesion to membranes and altering their permeability or ion transport properties, disturbing cellular phosphate management, and inhibition of DNA synthesis and DNA damage by breaking the H-bonding; crumpling proton pump; ROS generation; denaturation of ribosome; and inactivation of proteins and enzymes by bonding on active sites (Moreno-Garrido et al., 2015; Kwok et al., 2016; Taylor et al., 2016). Smaller AgNPs (<80 nm) are shown to be able to enter into bacterial cells (Klaine et al., 2008), but there are contradictions on the entry of bigger NPs into the cells of different algae (He et al., 2012). Several reports have indicated the “Trojan horse” effect of AgNPs in which NPs start releasing ionic Ag^+^ after its entry and damage the cellular structure (Huang et al., 2016). However, due to the ability of some microalgae to produce internal NPs from dissolved metals (Moreno-Garrido et al., 2015), the intracellular NPs observed in certain studies should be carefully investigated.

#### Tolerance Mechanism

Algal cells have specific mechanisms to cope up and reduce the toxic effects produced by AgNPs. Algal cell egests certain compounds to tolerate the toxic effects of AgNPs. The discharge of metal chelates from root system may either repress the availability of toxic metal ions excreted through AgNPs or increase its intake of metals (Dong et al., 2007; Navarro et al., 2008). These excreted compounds may regulate the dissolution rate of metals released from AgNPs. Certain compounds released from algal cells may also increase the AgNP flocculation and repress its bioavailability (Soldo et al., 2005). Many exopolymeric substances are released upon the introduction of AgNPs into the cell and this lead to their detoxification mechanisms (Miao et al., 2009).

Although AgNPs affect the algal population, these algal populations can also affect the potential toxicity and release of Ag from AgNPs by producing extracellular dissolved organic carbon compounds (DOCs) in order to inactivate AgNPs toxicity (Taylor et al., 2016; Xu et al., 2016). Hence, this is certain that feedback response by the algae against the presence of NPs seems to occur in the cells which can alter bioavailability and chemical behavior of the NPs (He et al., 2012). Therefore, it may be understood that algal species have various tolerance mechanisms for the initial impacts posed by the AgNPs while concentration and exposure duration are the significant factors determining the longevity of the effects and also their intensities on the algal species. However, it still seems a bit complex in the arena of research to understand comprehensive tolerance mechanisms in algal cells possibly due to greater diversity in them and complex ecological conditions in which they live which, further, have certain effects on the adaptation and tolerance mechanisms of the algal cells toward AgNPs.

### Interaction with Plants

Plants as producers are the building blocks of the basic structure of any ecosystem. Plants uptake, translocate and accumulate AgNPs from their surrounding growing medium (Monica and Cremonini, 2009). When AgNP is released in the environment, they find their way into the plants through food chain and then impart toxicity to them. Various studies show marked positive and negative impacts of AgNPs on plants (Siddiqui et al., 2015; Tripathi et al., 2017b) which depend on various factors regulating the uptake and accumulation in plants (Wang et al., 2015). Uptake of AgNPs depends upon the cellular permeability of the concerned plant and also on the different size and shape of AgNPs (Tripathi et al., 2017b). After entry into cells and sub cells, they create biological alterations and essential macrobiotic elements such as protein are affected (Griffitt et al., 2009; Pham et al., 2012). After their entry into the roots, AgNPs have been found to regulate the accumulation of protein, such as CDK-2 (cell division cycle kinase-2), 1,6-bisphosphate aldolase, protochlorophyllide oxidoreductase (Siddiqui et al., 2015). They also regulate the expression of some genes involved in cellular metabolism such as expression of IAA-8 (Indole acetic acid protein 8), RD22 (dehydration responsive), and NCED3 (9-*cis*-epoxycarotenoid dioxygenase) (Siddiqui et al., 2015). In *Arabidopsis thaliana*, inhibition of root elongation of seedling by activation of ACC (aminocyclopropane- 1-carboxylic acid) declines the expression of aminocyclopropane- 1-carboxylic acid synthase 7 and aminocyclopropane- 1-carboxylic acid oxidase 2, that ultimately inhibits biosynthesis of ethylene under the effect of AgNPs uptake in roots (Siddiqui et al., 2015). AgNPs also affect plants by producing ROS together with DNA destruction (Roh et al., 2009; Kim et al., 2013).

#### Uptake, Translocation, and Accumulation

**FIGURE 2:**
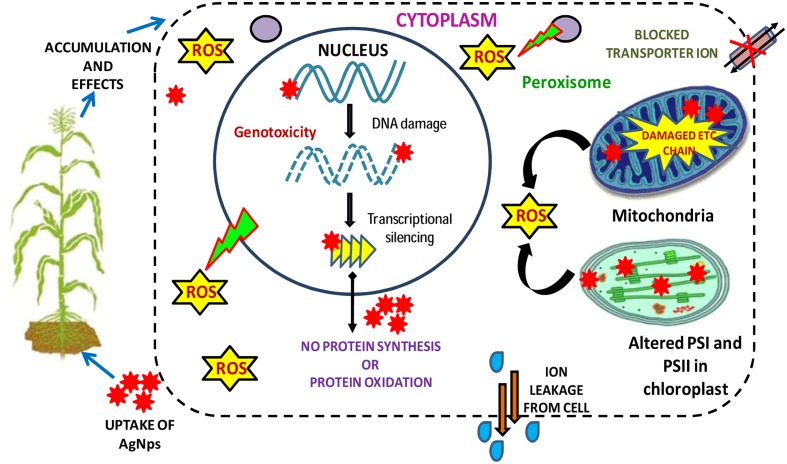
**Figure showing the major phytotoxicity of AgNPs occurring on various cell organelles of a plant cell and consequently on their metabolism (modified from Ma et al., 2013)**.

Plant cell walls are mainly composed of cellulose which act as semi-permeable layer precisely permitting the entry of smaller particles and inhibiting the larger ones. The cell wall of the root cells is the main site through which AgNPs enter in plant cells (**Figure 2**). After entering into the plant, they penetrate the cell wall and plasma membranes of epidermal layer of roots, and then enter inside the vascular tissues (**Figure 2**). The AgNPs come in the plant together with the plant’s uptake of water and other solutes. The cell wall consists of pores which are smaller than the NPs (Ma et al., 2010) and the cell wall serve as natural sieves (Navarro et al., 2008). The small sized AgNPs transit through the pores and enter into plasma membrane whereas large sized AgNPs are sieved out. They are further translocated to the stems and then to the leaves.

Sometimes, AgNPs influence the creation of new pores which permits the internalization of large AgNPs through cell wall (Navarro et al., 2008). Large leaf area and static plants enhance the accumulation of AgNPs from the ecosphere (Dietz and Herth, 2011). Translocation of AgNPs is aided by endocytosis (Ovećka et al., 2005; Fabrega et al., 2010) which include the creation of vesicle that enfold the material and finally transport AgNPs from plasma membrane to the cells. The AgNPs that eventually reach to the cell wall may also be translocated through plasmodesmata (Heinlein and Epel, 2004; Lucas and Lee, 2004; Ma et al., 2010). Plant’s acquirement of AgNPs usually occur via intercellular spaces and translocated within the cells of plant through the plasmodesmata process. After getting accumulated in the plant cells, AgNPs pose many gregarious impacts on plants including physiological, biochemical, and structural as well (Tripathi et al., 2017b).

#### Toxicity

Silver nanoparticle causes phytotoxicity in plants to a great extent which can be observed variably by analyzing different physical, physiological, biochemical, and structural traits (Tripathi et al., 2017b) (**Table 2**). They damage the cell membranes; interrupt ATP production as well as DNA replication (**Figure 2**). The enhanced production of ROS and subsequent generation of oxidative stress lead to various toxic impacts and may also affect the gene expressions and the demolition of DNA due to enhanced generation of ROS. Toxicity of AgNPs can be seen from seedling growth stage up to a full developed stage of the plants (Yin et al., 2012). It generally gives negative impact on the root growth of germinating seedlings and reduces the fresh biomass of the plant through reduction in root elongation and weight (Tripathi et al., 2017b). They also induce morphological modifications not only on the contact parts of the roots but also in the stem and leaves (Tripathi et al., 2017b). AgNPs modify the expression of several proteins of primary metabolism and cell defense system (Ma et al., 2010). AgNPs also affect the reproductive structure of the plant and destruction of DNA involve the creation of chromatin bridges, stickiness, disarranged metaphase and multiple chromosomal breaks (Panda et al., 2011; Patlolla et al., 2012; Anjum et al., 2013).

**Table 2:** Impact of different concentration of AgNPs in plants.

Plants	Size	Concentration	Inimical effects	Reference
*Cucurbita pepo*	>100 nm	500 mg L^-1^	Rate of transcription declined up to 66–84%. Biomass reduction was also reported	Musante and White, 2012
*Triticum aestivum*	10 nm	0–5 mg kg^-1^	Reduction in root and shoot length occur in dose dependent manner	Dimkpa et al., 2013
*Triticum aestivum*	10 nm	0–5 mg kg^-1^	Accumulation of oxidized GSSG in dose dependent manner	Dimkpa et al., 2013
*Cucurbita pepo*	NA	250 and 750 mg L^-1^	49–91% decreased rate of transpiration and biomass as compared to silver compound	Hawthorne et al., 2012
*Cucumis sativus; Lactuca sativa*	2 nm	62, 100, and 116 mg L^-1^	Negotiable toxicity	Barrena et al., 2009
*Linum usitatissimum*	20 nm	20, 40, 60, 80, and 100 mg L^-1^	No effect seen on germination	El-Temsah and Joner, 2012
*Lolium perenne*	0.6–2 nm (Colloidal)	10 mg L^-1^	20% reduction in germination percentage	El-Temsah and Joner, 2012
*Lolium perenne*	0.6–2 nm (Colloidal)	20 mg L^-1^	50% reduction in germination percentage	El-Temsah and Joner, 2012
*Lolium perenne; Linum usitatissimum*	0.6–2 nm (Colloidal)	10 mg L^-1^	Reduction in length of shoot	El-Temsah and Joner, 2012
*Hordeum vulgare; Lolium perenne; Linum usitatissimum*	0.6–2 nm (Colloidal)	20 mg L^-1^	Reduction in length of shoot	El-Temsah and Joner, 2012
*Hordeum vulgare*	5 nm	10 mg L^-1^	Reduced rate of germination	El-Temsah and Joner, 2012
*Linum usitatissimum; Hordeum Vulgare*	5 nm	10 mg L^-1^	Reduction in length of shoot	El-Temsah and Joner, 2012
*Hordeum vulgare*	20 nm	10 mg L^-1^	Reduction in rate of germination and shoot length	El-Temsah and Joner, 2012
*Hordeum vulgare; Lolium perenne*	20 nm	20 mg L^-1^	Declined shoot length	El-Temsah and Joner, 2012
*Cucurbita pepo*	100 nm	100, 500, and 1,000 mg L^-1^	41–79% of reduction in rate of transpiration	Stampoulis et al., 2009
*Lolium multiflorum*	6 nm (Gum arabic-coated	1–40 mg L^-1^	Dose dependent toxicity Undeveloped root hairs Crumpled cortical cells Ruptured epidermis Undeveloped root cap Declined biomass Decreased root length	Yin et al., 2011
*Populus deltoides nigra*	25 nm	100 mg L^-1^	87% declined evapotranspiration that result in decreased fresh biomass of leaves, stem, and roots.	Wang et al., 2013
*Arabidopsis thaliana*	5 and 10 nm	1 mg L^-1^	Growth of root completely inhibited	Wang et al., 2013
*Oryza sativa*	NA	1,000 mg L^-1^	Vacuolar damage in root cells Cell wall breakage	Mazumdar and Ahmed, 2011
*Allium cepa*	70 nm	0–80 mg L^-1^	Cytotoxicity seen at LC_50_, i.e., up to 10 mg L^-1^ concentration DNA damage at 10 mg L^-1^ concentration	Panda et al., 2011
*Allium cepa*	24–55 nm	0–80 mg L^-1^	Generation ROS that causes damage in structure of DNA and ultimately death of the cell	Panda et al., 2011
*Allium cepa*	<100 nm	100 mg L^-1^	Sticky chromosomes led to chromosome breakage and disturbance in metaphase, that result in disruption of cell wall	Kumari et al., 2009
*Vicia faba*	60 nm	12.5, 25, 50, and 100 mg L^-1^	Increased chromosomal aberrations	Patlolla et al., 2012

Silver nanoparticles also affect the photosynthetic system of the plants (Tripathi et al., 2017b) through reducing total chlorophyll, affecting fluorescence parameters, and enhancing proline content (Monica and Cremonini, 2009). The main reason behind the dreadful toxicity of AgNPs in the plants is its impact on the biochemical properties of plants and inducing free radical generation resulting in induced oxidative stress in plant cells (Nair et al., 2010). The increased generation of the hydrogen peroxide (H_2_O_2_) in the plants cells is also an important toxic effect to be considered which affect the growth and development of the plants and kill the cells (Monica and Cremonini, 2009; Tripathi et al., 2017a,b). AgNPs may also affect the mitochondrial membrane potential (DWm) of roots with increasing concentrations (Hsin et al., 2008). The toxicity of AgNPs is more noticeable in roots as compared to shoots because roots are the main site of interaction while plant’s self-defense mechanism involve translocation of the AgNPs from roots to shoots and thus restrict its accumulation in above ground parts completely or partially (Yin et al., 2012; Vannini et al., 2014). The research is needed to understand the effects of NPs on cellular level and how to reduce NPs’ inherent toxicity by modifying some cellular processes. One way could be the modification in the osmolyte concentration in the environment for which researches should concern for plasmonic NP–cell interaction and internalization dealing with the NP surface composition and aggregation behavior in the cellular environment (Siddhanta et al., 2016). Some researchers have shown the osmolyte-based approach to reduce the toxicity of NPs by surface aggregation on the plasma membrane of the cells without changing the specific surface functionalization. The toxicity may also be reduced by inhibiting protein aggregation through lysozyme–AgNP interaction (Siddhanta et al., 2015).

#### Tolerance Mechanism

The toxicity of AgNPs leads to the cellular damage as well as affects metabolic activities which lead to the phytotoxicity in plants. Thus, activation of tolerance mechanism is very important so that the plant cells should be protected from stress conditions. The different stresses of plant cells require varied tolerance mechanisms to eliminate their toxic effects. The enhanced concentrations of cellular metabolite proline as well as oxidative stress controlling genes indicates the readiness of plant’s antioxidative defense mechanism for the termination of oxidative stress factors (Apel and Hirt, 2004; Nair et al., 2010). According to Hsin et al. (2008), the cells should be given pretreatment of cyanide which suppresses the mitochondrial electron transferring process of cytochrome C oxidase that intercepts the generation of ROS through AgNPs. For the protection of cells against induced generation of ROS, plants involve many processes such as regulation of genes in which oxidative stress responses lead to the production of antioxidant enzymes (Apel and Hirt, 2004). There are various types of enzymatic scavengers present in cells of plants such as SOD, CAT, and APX which are ready to protect the cells from stress conditions (Nair and Chung, 2014). These toxic effects are dependent on various factors of plants, i.e., species, seeds, seedlings, and cell suspensions; and AgNPs, i.e., its concentration, size, aggregation, and functionalization. Also, the surrounding factors like temperature, time, and method of exposure can inhibit the AgNP phytotoxicity (Navarro et al., 2008; Siddhanta et al., 2016).

### Interaction with Microbes

Microbes include bacteria, molds, yeasts, and viruses that are present in the environment and may induce several diseases. All having a very simple morphological structure perform different types of metabolic functions. For studies regarding AgNPs and their interaction with microbes, bacteria are among the most important organisms due to their small size and simple cell structure (Pal et al., 2007; Samberg et al., 2011; Prasad et al., 2016). As they are pathogenic in nature and result in serious infections for all life forms, a new antimicrobial agent is required to suppress the formation of pathogens. Silver compounds have been used as an inorganic antimicrobial agent to combat contagion of different pathogens since ancient days (Shrivastava et al., 2007; Lee et al., 2007). AgNPs act as an antibacterial agent toward bacterial stresses and eliminate its atrocious effects (Lee et al., 2007). Studies have also been conducted on the interaction of AgNPs with fungi and viruses and they have also been found to be affected by AgNPs at various concentrations (Velmurugan et al., 2009; Galdiero et al., 2011).

#### Uptake, Translocation, and Accumulation

Beside the simple shape or structure of bacteria, they possess a well developed structure of cell that performs many biological functions. Intracellular distribution of any solute or AgNP depends on their surface area to volume ratio (Pal et al., 2007). Studies have demonstrated that some small granular (electron dense) structures either accumulate in the cells or adhere near the cell wall (Feng et al., 2000). Furthermore, Feng et al. (2000) also demonstrated that accumulation of the sulfur, silver ions and dense electron granules occurs in the cytoplasm. This process disrupts the bacterial membrane and makes the entrance of AgNPs in the cell easy. Moreover, it also leads to the alteration in integrity of cell by continuous leakage of intracellular potassium from the cell (Navarro et al., 2008). The probable mechanism for the target and interaction of silver species might also be the thiol groups found in proteins (Lok et al., 2006). Similarly, another site for interaction of silver species to the bacterial cell could be phospholipid membrane (Lok et al., 2006). In the same way, fungal cells comprise of cell wall which inhibit the transition from AgNP in cells. Fungi cell wall consists of some significant constituents like carbohydrates which form a stiff and elusive structure (Navarro et al., 2008). The main component of fungal cell is their chitinous cell wall that is semipermeable in nature and acts as a sieve to allow the transition of small particles while inhibiting the larger ones. However, sometimes the pore size increases during reproduction period due to the effect of AgNPs and recently formed pores permit the translocation of the larger AgNPs (Ovećka et al., 2005; Navarro et al., 2008). Due to substantial alteration during exposure of AgNPs, “pits” are formed on the cell wall surface leading into the creation of pores and result in the destruction of cell metabolism (Navarro et al., 2008). The membrane barriers may collapse due to AgNPs by outflow of ions and other materials which alters the electrical potential of the membrane.

#### Toxicity

**FIGURE 3:**
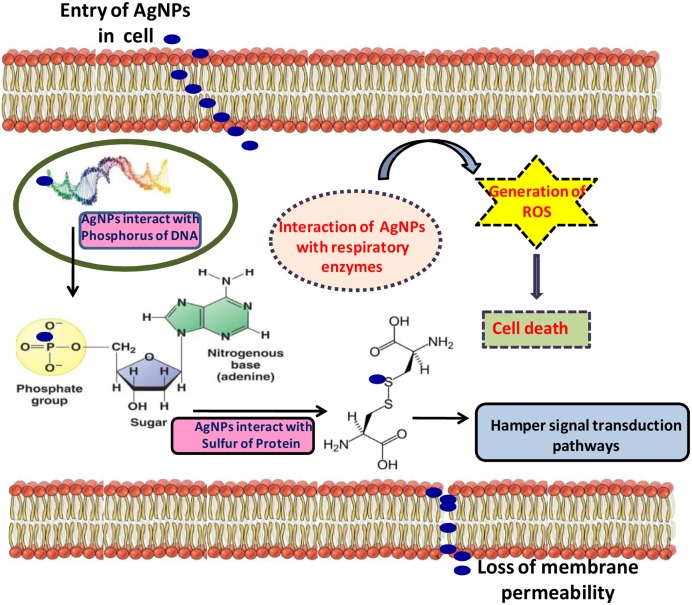
**Figure showing the inimical toxicity of AgNPs in the bacterial cell (modified from Prabhu and Poulose, 2012)**.

Silver ions and the related compounds show high toxicity to the microorganisms (**Table 3**). Choi et al. (2008) described the inhibitory mechanism of silver or the toxicity created by silver in microbes and in the context of the toxicity of AgNPs, free silver ions were found to be more toxic than silver nitrate. Moreover, when silver ions react with SH functional group of proteins, they cause inactivation in bacterial cell (Morones et al., 2005). Also, concentration of silver ions in micromole level has been found to inhibit the process of microbial respiration by uncoupling the electrons involved in phosphorylation and thus disrupt the permeability of membrane (Feng et al., 2000). Both Gram-negative (*Escherichia coli*) and Gram-positive (*Staphylococcus aureus*) types of the bacteria are found to be affected by the silver ions (Feng et al., 2000; Jung et al., 2008). AgNPs may interact with nucleic acid and lead to the destruction in DNA replication in bacteria (Feng et al., 2000) (**Figure 2**). From many studies and researches, it is proved that AgNPs or silver ions may be toxic to some species of bacteria like *E. coli* (Gogoi et al., 2006; Kim et al., 2007; Mohan et al., 2007) and yeast (Kim et al., 2007). It was also found that after interaction of AgNPs with *E. coli*, the membrane integrity completely disrupts due to high surface area to volume ratio of AgNPs facilitating more interaction with *E. coli* (Raffin et al., 2008). However, Morones et al. (2005) demonstrated that penetration of AgNPs in Gram-negative bacteria (*Vibrio cholera*, *Salmonella enterica typhi*, *E. coli*, and *Pseudomonas aeruginosa*) depend on their size. The most preferable size they observed was in between 1 and 10 nm. The proteomic analysis in *E. coli* revealed the change in expression of HSPs (heat shock proteins) due to the impact of AgNPs (Lok et al., 2006) which disrupts the bacterial membrane and entrance of the smaller particles in the cell membrane becomes easy. This process also leads to the alteration in cellular integrity by continuous leakage of intracellular potassium from the cell which reduces the ATP and damages the cell viability. A hypothetical toxicity mechanism has been given in **Figure 3**.

**Table 3:** Effect of different concentrations of AgNPs on microbes.

Microbes	Size	Concentration	Effect	Reference
*Escherichia coli*	12 nm	50–60 μg cm^-3^	Inhibition of bacterial growth Increased permeability due to formation of “pits”	Sondi and Salopek-Sondi, 2004
*Aspergillus* sp.	30–45 nm	50 μg mL^-1^	AgNPs shows antifungal activity Suppress the growth of fungal cells	Kuppusamy et al., 2015
*Pneumocystis* sp.	30–45 nm	50 μg mL^-1^		
Yeast	13.5 nm	13.2 nM	Generation of free radicals Loss in permeability of membrane	Kim et al., 2007
*Escherichia coli*	13.5 nm	3.3 and 6.6 nM		
*Staphylococcus aureus*	13.5 nm	>33 nM		
*Escherichia Coli*	3 nm	40–140 μg mL^-1^	Inhibitory effect	Ruparelia et al., 2008
*Bacillus subtilis*	3 nm	40 μg mL^-1^		
*Staphylococcus aureus*	3 nm	120 μg mL^-1^		
*Escherichia coli*	40 nm	40 μg mL^-1^	On interaction of bacterial cell with AgNPs causes Proton Motive Force dissipation and finally death of the cell	Yoon et al., 2007
*Bacillus subtilis*	40 nm	20 μg/mL		
*Escherichia coli*	10 nm	0.1–1 mg L^-1^	Damage occur in protein and membranes	Hwang et al., 2008
*Escherichia coli*	From 39 to 41 nm	0.1–10 μg mL^-1^	Truncated AgNPs possess biocidal effect	Pal et al., 2007
*Escherichia coli*	9.3 ± 2.8 nm	0.4–0.8 nM	Unstable outer membrane Disintegrated plasma membrane	Lok et al., 2006
*Escherichia coli*	9.3 ± 2.8 nm	0–100 μg mL^-1^	Small sized AgNPs showed more detrimental effect than larger ones	Lok et al., 2006
Nitrifying bacteria	21 nm	0.05–1 mg L^-1^	Generation of Reactive Oxygen Species	Choi et al., 2008
Autotrophic nitrifying bacteria	14 ± 6 nm	1 mg L^-1^	Respiration declined by 87% (calculated)	Choi and Hu, 2008
*Pseudomonas fluorescens*	65 ± 30 nm	0–2000 ppb	Toxicity of AgNPs varies according to the pH	Fabrega et al., 2009
*Pseudomonas putida* biofilm	65 ± 30 nm	0–2000 ppb	Toxicity of silver nanoparticles enhanced in combination of organic matter	Fabrega et al., 2010
*Escherichia coli*, *Staphylococcus aureus*	26 nm	MIC range of 1.69–6.75 μg mL^-1^	Enhanced antibacterial activity	Kvitek et al., 2008
*Escherichia coli, Salmonella typhi*, *Pseudomonas aeruginosa* and *Vibrio cholerae*	16 ± 8 nm	0–100 μg mL^-1^	AgNPs of less than 10 nm attached with membrane and cause toxicity	Morones et al., 2005
*Escherichia coli*, Ampicillin-resistant *Escherichia coli*, Multi-drug resistant *Salmonella typhi*, *Staphylococcus aureus*.	10–15 nm	5–35 μg mL^-1^	More detrimental for Gram-negative bacteria as compared to Gram-positive.	Shrivastava et al., 2007

While, in fungal cell wall, formation of new larger pores takes place due to effect of AgNPs and thus the transition of large AgNPs becomes easier. The perturbation of membrane by AgNPs leads to the generation of glucose and trehalose which indicate that they are the intracellular components of the membrane (Siddhanta et al., 2016). Reidy et al. (2013) have reported the mechanisms involved in antimicrobial actions of AgNPs which start with the adhesion of AgNPs on the surface of bacterial cell and changes the properties of membrane. AgNPs (smaller than 5 nm) have also been reported to suppress the growth of nitrifying bacteria (Choi and Hu, 2008; Reidy et al., 2013). After the disintegration of AgNPs, the released Ag ions interact with bacterial cell wall which chiefly consists of sulfur protein resulting in compromised functionality (Levy and Marshall, 2004; Reidy et al., 2013). Silver ions also interact with the cytoplasmic proteins of bacterial cell wall (Cao and Liu, 2010; Reidy et al., 2013) and also affect the thiol group leading into the improper functioning or inhibition of bacterial cell. Uptake and accumulation of silver ions in bacterial cell disrupt hereditary biomolecules such as DNA and may lead to many unwanted changes in the genetic makeup of the bacteria (Feng et al., 2000).

#### Tolerance Mechanism

Bacterial cells also adopt some defense mechanisms to save themselves from the harmful effects of AgNPs. It has been reported that the peptidoglycan membrane thickness and their component (phospholipids) participate in the defense mechanism against AgNPs as their first line of defense (Sedlak et al., 2012). In order to protect bacterial cells from the toxic effects of AgNPs, many proteins such as heat shock proteins also get activated (Sedlak et al., 2012). Generally, a bacterium shows tolerance mechanism against the high concentrations of AgNPs, and preferably use eﬄux pumps resistant toward silver ions in natural environment (Jung et al., 2008). The encoding of this eﬄux pump is carried out by the plasmid-borne cassettes and it can also transmit to other strains of bacteria. Beside this, for the production of periplasmic silver ion-binding protein along with pumps responsible for eﬄux of ions, i.e., P-type ATPase and chemiosmotic silver ion/H^+^ exchange protein, a sensor or transcriptional regulatory system play a key role behind it (Kvitek et al., 2008). According to Khan et al. (2011), *Bacillus pumilus* is tolerant toward AgNPs’ antibacterial activity at high concentrations. The growth of bacteria stays the same while the diminution of extracellular polymeric substances (EPS) has been recorded (Taylor et al., 2016). According to Feng et al. (2000) and Jung et al. (2008), both types of Gram-negative (*E. coli*) and Gram-positive (*S. aureus*) bacteria accumulate dense electron light particles or granules as a defense strategy in the center of the cell. This region actually consists of thicker DNA (deoxyribonucleic acid) molecules and this thickness provides them security against the attack of silver ions.

Jung et al. (2008) also reported that in the presence of silver ions, some morphological changes also occur in the bacterial cells and these bacterial cells attain a non-culturable position and at last, lead to death. The peptidoglycan layer in Gram-positive bacteria is also very important in providing protection against AgNPs due to their thickness and shows a high degree of inhibitory effect against the adverse effects of silver ions, especially in *E. coli* (Jung et al., 2008). Bacterial defense mechanism also works even at molecular level (Silver, 2003). After the exposure of bacteria against the silver salts or silver ions, genes of plasmid and chromosomes have shown high degree of resistance mechanism (Silver, 2003). However, studies have been able to solve some of the questions relating to the defense mechanisms against AgNPs in the bacterial cells, such explorations regarding other microbial forms are still sporadic and a great deal of work related to genetic transformations and molecular markers in these microbes is yet to be done.

## Conclusion and Future Research Approaches

It is quite evident that the technological interventions related to the nanotechnology have immense use and importance in modern times; however, somehow they are leading to the destruction or imbalance in ecosystem with their unregulated release posing toxic impacts on plants, algae and micro-organisms. Although researches are being carried on the beneficial as well as harmful impacts of AgNPs, there is a need to work in order to understand the toxicity of AgNPs at the cellular level of these organisms and their further impacts. Studies have yet not been able to fix any conclusive results on the effective/lethal/sub-lethal/optimum concentrations of NPs/AgNPs as a whole or/and the organism wise on which some regulatory framework can be made. The data regarding this are inadequate and researches must be carried on looking these things into consideration. The toxicity of AgNPs is translocated from plants to other communities through food chain and leads to the disruption of balanced ecosystem. However, the food chain analysis and health effects on trophic structure on this regard is sporadic and must be considered in the studies. The differences existing among experimental results of toxicity are thus creating problems in interpretation of the toxicological data. The studies on the toxicity and tolerance in plants, algae and microbes on molecular level are yielding some good results though, studies regarding fungi, yeasts, and viruses are very few on these aspects.

There must be some microcosm studies involving ecosystem based studies on this regard. The molecular marker approach, the evaluation of the tolerance mechanism and their use to develop artificial tolerance in the organisms may pave the significant pathways in this research field not only for developing new nanomaterials of use but also for formulating some regulatory concentration in various components of the environment. There is a strong need for the appropriate association between analytical techniques and toxicological studies through which more understanding towards this subject may be developed for the future research projects. Studies are generating good amount of data to be interpreted. A common research platform is needed to agglomerate all the data and to reach out to a logical conclusion to safeguard the ecosystem functioning and humankind as well.

## Author Contributions

DKT, AT, S, ShS, KV, GY, RM, and YS designed the manuscript. DKT, AT, S, KV, and SwS wrote the manuscript. DKT, DC, ShS, VKS, YL, RU, and ND critically evaluated the manuscript.

## Conflict of Interest Statement

The authors declare that the research was conducted in the absence of any commercial or financial relationships that could be construed as a potential conflict of interest.
